# Design, Analysis, and Testing of a Type V Composite Pressure Vessel for Hydrogen Storage

**DOI:** 10.3390/polym16243576

**Published:** 2024-12-21

**Authors:** Maria Mikroni, Grigorios Koutsoukis, Dimitrios Vlachos, Vassilis Kostopoulos, Antonios Vavouliotis, George Trakakis, Dimitrios Athinaios, Chrysavgi Nikolakea, Dimitrios Zacharakis

**Affiliations:** 1 Adamant Composites Ltd., Agias Lavras & Stadiou, 26504 Patras, Greece; koutsoukis@adamant-composites.com (G.K.); vlachos@adamant-composites.com (D.V.); vavouliotis@adamant-composites.com (A.V.); trakakis@adamant-composites.com (G.T.); dimitrisath1@gmail.com (D.A.); nikolakea@adamant-composites.com (C.N.); zacharakis@adamant-composites.com (D.Z.); 2 Department of Mechanical Engineering & Aeronautics, Laboratory of Applied Mechanics and Vibrations, University of Patras, 26504 Patras, Greece; kostopoulos@mech.upatras.gr

**Keywords:** hydrogen storage, first ply failure (FPF), last ply failure (LPF), hydraulic burst pressure test, numerical model validation

## Abstract

Hydrogen, as a zero-emission fuel, produces only water when used in fuel cells, making it a vital contributor to reducing greenhouse gas emissions across industries like transportation, energy, and manufacturing. Efficient hydrogen storage requires lightweight, high-strength vessels capable of withstanding high pressures to ensure the safe and reliable delivery of clean energy for various applications. Type V composite pressure vessels (CPVs) have emerged as a preferred solution due to their superior properties, thus this study aims to predict the performance of a Type V CPV by developing its numerical model and calculating numerical burst pressure (NBP). For the validation of the numerical model, a Hydraulic Burst Pressure test is conducted to determine the experimental burst pressure (EBP). The comparative study between NBP and EBP shows that the numerical model provides an accurate prediction of the vessel’s performance under pressure, including the identification of failure locations. These findings highlight the potential of the numerical model to streamline the development process, reduce costs, and accelerate the production of CPVs that are manufactured by prepreg hand layup process (PHLP), using carbon fiber/epoxy resin prepreg material.

## 1. Introduction

**Figure 1:**
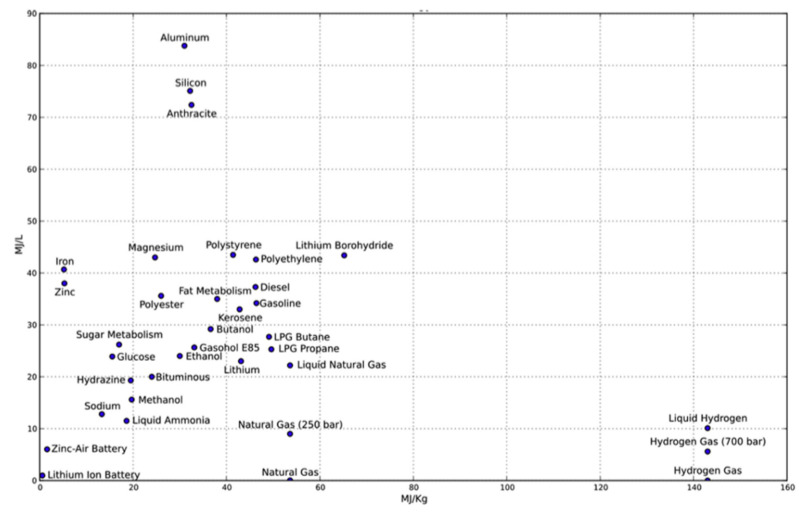
Comparison of energy densities by weight (MJ/kg) versus volume (MJ/L) for many common fuels and other useful materials [4].

The 21st century’s energy challenges demand urgent advancements in sustainable systems, with rising global temperatures intensifying the need for designs that prioritize environmental impact, lifecycle efficiency, and low-emission technologies [1,2]. In response to these challenges, researchers are exploring sustainable energy solutions. Hydrogen stands out for being a high-performance and environmentally friendly fuel [3]. It is considered non-toxic, colorless, and the most abundant element, offering significant benefits, including the highest gravimetric energy density compared to standard fuels, as Figure 1 shows. This means it can store large amounts of energy within a small mass [1]. However, hydrogen’s low volumetric energy density necessitates substantial storage space, as large amounts of energy need large space to be stored, posing significant technical and engineering challenges.

The solution to the hydrogen storage issue is tackled by developing pressure vessels, which are designed to safely withstand the internal pressure of compressed, liquid, or cryo-compressed hydrogen fuel throughout the vessel’s entire lifecycle [5]. These vessels must balance safety with minimizing weight to prevent additional fuel consumption. Moreover, to remain feasible and competitive, the cost of these vessels must be kept as low as possible [3].

**Figure 2:**
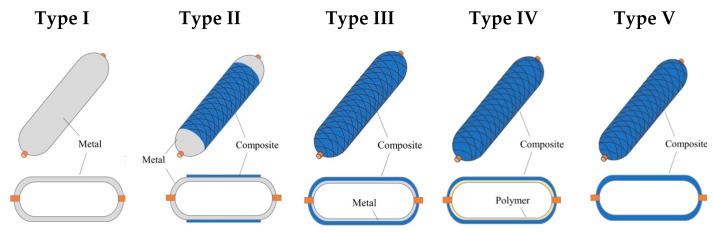
Different types of pressure vessels [2,14].

Thus, with the growing popularity of advanced hydrogen solutions, research and development efforts are focused on finding cost-effective, lightweight materials and innovative designs that enhance the performance of hydrogen storage tanks. Currently, pressure vessels, in the general market, are categorized into five main types, classified numerically from Type I to Type V, as presented in Figure 2. Each type represents a distinct approach to hydrogen storage, with differences in weight, cost, and material composition.

Type IV vessels are the most used in hydrogen storage applications, as extensive literature demonstrates their reliable performance in meeting pressure requirements. For example, Madhavi [6] presents a design of a composite pressure vessel with a length of 673.2 mm (pole to pole), a diameter of 350 mm, and a pole opening diameter of 160 mm, with a maximum expected operating pressure (MEOP) of 9.6 MPa (96 bar). Guillon et al. [7] outline verification tests for baseline metrics of a Type IV vessel, showing that it can withstand up to 225% of its service pressure, which corresponds to 1575 bar for the widely adopted 700-bar service pressure.

However, the current state-of-the-art pressure vessel technology, known as Type V, marks a significant advancement by eliminating the internal polymer liner used in Type IV vessels. Instead, Type V vessels rely entirely on carbon fiber laminates to provide both structural integrity and gas leakage [8]. This design offers the lightest possible solution and holds the potential to reduce both manufacturing costs and operational risks [3,9].

Research on Type V vessels began in the aerospace industry in the 1980s. Recent studies have focused on the design and manufacturing of Type V composite pressure vessels (CPVs), with several groups developing these tanks for commercial and industrial applications [8]. Automotive and bus applications typically use vessels with service pressures of 350 or 700 bar, while many industrial tanks operate at 200 bar [10]. Kothali et al. [11] designed a Type V CPV and developed a numerical model burst pressure calculated at 170 bar. Tian et al. [12] developed a vessel where matrix cracking occurred at 7 bar, but carbon fibers remained intact until burst pressure reached 30 bar. Air et al. [8] demonstrated full-scale Type V tanks tested to 41.4 bar and found that both the first ply failure (FPF) and burst occurred at lower pressures in linerless tanks compared to lined tanks.

To achieve improved performance, there is a need for better design and new material systems that are both lighter and capable of withstanding the required pressures. In addition to enhancing performance, accurate prediction of the vessel’s pressure limits is crucial for optimizing the design. Tian et al. [12] presented a filament-wound pressure vessel for a solid rocket motor, where the predicted burst pressure (80 bar) deviated by 3.75% from the experimental value (77 bar). Lin et al. [13] reported a 5.4% average difference between predicted and experimental burst pressures, demonstrating the effectiveness of the proposed method for predicting progressive damage in composite pressure vessels. Similarly, Rafiee and Torabi [14] observed deviations of 13%, 17.5%, and 15.9% between experimental and theoretical predictions for vessels with different stacking sequences. The authors claim that these deviations are acceptable, emphasizing that factors such as modeling approaches, selection of failure criteria, manufacturing inconsistencies, and the inherent stochastic nature of composite materials can significantly contribute to these discrepancies.

**Figure 3:**
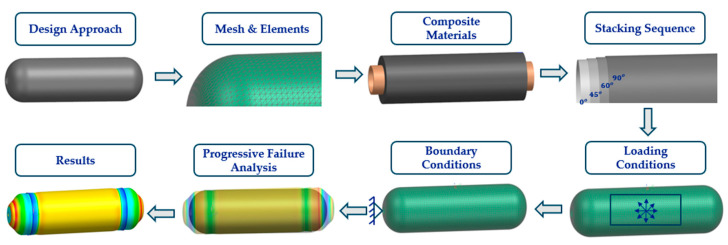
Process steps for the development of the CPV numerical approach.

**Figure 4:**
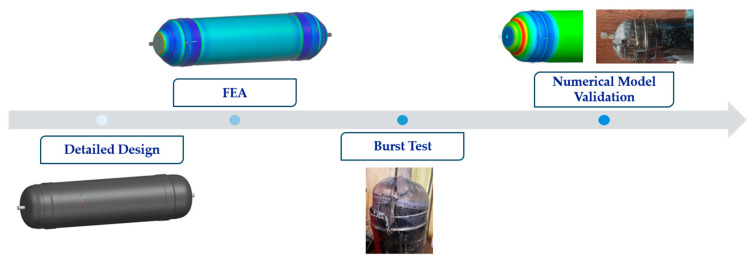
Schematic diagram with the objectives of this study.

Despite significant advancements in the research and development of pressure vessels, the peer-reviewed literature on accurately predicting the performance of Type V CPV remains limited, highlighting the need for further comprehensive studies. This research aims to address this gap by enhancing the reliability of CPVs through a more in-depth understanding of their design, analysis, manufacturing, and testing processes, by designing a CPV that can store liquid hydrogen and can withstand an operational pressure of 60 bar. The primary objective is to develop a numerical model of a Type V CPV using NX Siemens/Simcenter, capable of accurately predicting performance up to the point of burst. A mesoscale model will be employed to predict performance, focusing on degradation and failure at the ply level. As Figure 3 presents, the initial step involves defining the CPV geometry, creating the mesh, material properties, stacking sequence, and loading and boundary conditions. Using these inputs, a progressive failure analysis model is developed to predict numerical burst pressure (NBP), as Figure 4 shows, which is then compared to physical burst test results to validate the accuracy of the numerical model [15].

After developing the numerical model, the CPV is manufactured using a prepreg hand lay-up process (PHLP). Although automated fiber placement (AFP) and automated tape layering (ATL) are the most common manufacturing processes for Type V CPVs, PHLP was chosen due to its lower permeability issues and higher technology readiness level compared to AFP and ATL laminates [8]. Finally, as Figure 4 shows, to validate the numerical model, a hydraulic burst test is performed on the CPV, and the results are compared to the model’s predictions.

## 2. Materials and Methods

### 2.1. Numerical Model

#### 2.1.1. Design Approach

For Type II–IV tanks, the internal liner serves as the mandrel and remains within the vessel after the winding process is completed. However, for Type V tanks, the mandrel must be removed to achieve the linerless design. Research has shown that collapsible, soluble, or lost mandrels, which can be removed by dissolving with water, are viable options for these processes. For example, materials such as sand and plaster have been successfully used as mandrel materials [6]. However, as noted in [8], mandrel removal, particularly around the polar opening, remains a significant challenge, as removing the mandrel material without leaving any residue is critical. Azeem et al. [10] address these challenges by using a two-piece composite shell mandrel that can be removed before rebonding the halves. This method also simplifies the installation of internal components and inspection procedures.

**Figure 5:**
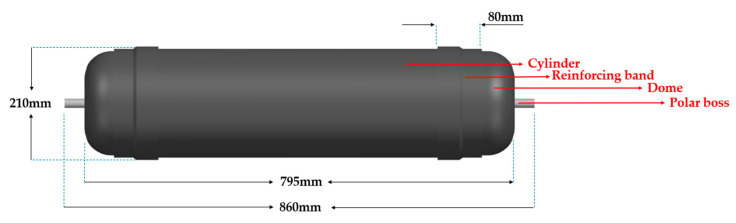
Basic dimensions and components of the CPV.

In this study, we adhere to this guidance as it also aligns with our decision to manufacture the vessel using PHLP. More specifically, the vessel designed for this study consists of seven assembled components: a cylinder, two end domes, two reinforcing bands, and two polar bosses attached to the pole openings, as illustrated in Figure 5. The lay-up process involves the use of two separate steel mandrels for the manufacturing of the domes and the cylinder and their curing under autoclave conditions (P = 7 bar, T=120 °C). The assembly plan entails joining the two domes with the cylinder and utilizing reinforcing bands at the overlap regions to enhance the longitudinal strength of the CPV.

Therefore, the design approach must carefully investigate the most effective methods for connecting these components. A thorough literature review, as documented in reference [16] and summarized in Table 1, identified various joint configurations suitable for connecting the domes with the cylinder. Among the configurations, Configuration 3 closely resembles Configuration 2 but with the distinction that the joints feature two interfaces. This enhancement improves the connection’s integrity, ensuring a tighter seal and preventing leakages. Thus, Configuration 3 was selected for this study. Consequently, the metallic molds for the cylinder and dome were designed to align the external surface of the domes with the internal surface of the cylinder. To further reinforce the longitudinal stiffness in the connecting areas, two carbon fiber-reinforced polymer (CFRP) bands were added as reinforcements on the external surface of the domes and the cylinder, ensuring a robust connection [6].

After outlining the assembly of the vessel components, it is essential to present the basic dimensions of the CPV used in this study. The vessel, as Figure 5 shows, has a total pole-to-pole length of 795 mm ($L_{vessel}$) and an outer diameter of 210 mm ($D_{vessel}$). To ensure that the pressure vessel can withstand the operating pressure, the composite thickness (t) is not uniform throughout the structure. In the critical connection areas, two reinforcing bands were incorporated, each with a geometry tangent to the dome-cylinder connection and a length of 80 mm ($L_{rb}$). Also, metallic bosses are attached at both polar openings, serving as connection points for these necessary valves and plugs, which are used for refueling the tank and extracting hydrogen during operation [17].

#### 2.1.2. Mesh and Elements

Selecting the appropriate mesh and element type poses a significant challenge, requiring a balance between computational efficiency and result accuracy. Various optimization methods have been reported in the literature, including the use of axisymmetric models that represent only part of the geometry [10]. However, this study will employ a complete model as the uneven overlaps of the domes cause misalignment of the reinforcing bands, leading to asymmetry and necessitating a full model analysis.

According to [10,18], different simulation types can be compared as follows:

1. Simulation Using 3D Elements
2. Simulation Using Conventional Shell Elements
3. Simulation Using Continuum Shell Elements
4. Simulation Using Mixed Methods

Conventional shell elements are often employed as they simplify the modeling process. Alternatively, shell elements based on three-dimensional cells offer a hybrid approach, combining the advantages of both conventional shell and solid models. The findings from this study indicate that continuum shell elements strike an optimal balance between accuracy and computational efficiency. However, given the presence of both composite and metallic parts in this study, a hybrid model incorporating both 2D and 3D elements is utilized [7]. For metallic components such as polar bosses, 3D elements are employed, as 2D elements are insufficient to accurately represent the complex geometry and thickness of these parts [10].

After determining that a hybrid approach utilizing both 2D and 3D elements will be implemented, it is crucial to establish appropriate mesh densities [10,18]. Coarse mesh may cause inaccurate results but finer meshes significantly increase computational demands, so it is vital to find the coarsest mesh that still provides sufficient accuracy [19,20]. Thus, in this study, for 2D surfaces such as cylinders, domes, and reinforcing bands, CQUAD4 elements are selected, while for 3D geometries such as polar bosses, the chosen element type is CTETRA10. Regarding the element size of every part, based on the mesh independence study, a 3mm element size is chosen for all parts. Using the same mesh size across all parts avoids the need for mesh transitions that would require triangular elements, which are generally not recommended due to their accuracy. As Table 2 shows, mesh sizes finer than 3 mm, such as 2 mm, significantly increase computational time by 4 times, while yielding only a 1.3% improvement in accuracy. Although coarser meshes, such as 5 mm, 6 mm, and 7 mm, offer faster computation times, the associated reduction in accuracy becomes more significant. Consequently, mesh sizes of 3 mm and 4 mm are the optimal choices, with 3 mm ultimately selected due to its favorable balance of computational efficiency and accuracy, as the additional time is not valuable.

#### 2.1.3. Composite Materials

The CPV analyzed in this study integrates two distinct materials: high-strength steel was used for the polar bosses, and composite material for the cylinder, domes, and reinforcing bands. The polar bosses are modeled as linear elastic and isotropic and composite layers are treated as orthotropic materials. As a prototype model, this study began with a comprehensive review of state-of-the-art composite materials presented in Table 3, identifying fiber and matrix combinations used in similar projects to guide material selection. As Air et al. refer [8], the majority of existing composite overwrapped pressure vessels (COPVs) and CPVs utilize carbon fiber reinforcement in combination with a thermoset matrix, due to its high strength, stiffness, ease of production, and reliability. Thus, the composite layers of the vessel analyzed in this study are fabricated from Carbon fiber/Epoxy resin (CF/E), a material whose properties at room temperature are presented in Table 4.

#### 2.1.4. Stacking Sequence

**Figure 6:**
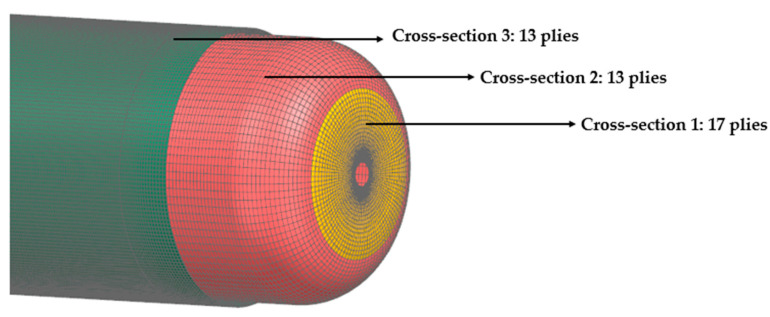
Different cross-sections of the tank.

To optimize the finite element model (FEM), various configurations were evaluated by adjusting the number and sequence of layers to meet the target operational pressure of 60 bar. As this vessel is a prototype and the primary objective at this stage was not to achieve an optimized stacking sequence, an initial layup plan was adopted considering the load bearing and manufacturability requirements. For the domes, as illustrated in Figure 6, three distinct cross-sections were designed. Cross-section 1 features a layup configuration of $\left[0/0/+45{}^{\circ}/\left(90/0/90/0/90/\bar{0}\right)s/-45{}^{\circ}/90/0\right]$, while cross-section 2 adopts a layup of $\left[{\left(0/90\right)}_{3}/\bar{0}\right]s$, with 0° plies oriented along the longitudinal axis. For the cylindrical section, a layup of $\left[0/+60/90/-60/90/90/\bar{0}\right]s$ was used. This differentiation in cross-sectional thicknesses, while potentially introducing regions susceptible to delamination, is specifically designed to enhance the structural integrity of the dome area, near the polar boss-dome interface. The addition of plies in cross-section 1 aims to reinforce this area, where higher stress concentrations are expected due to the significant stiffness mismatch between the steel and CFRP material.

**Figure 7:**
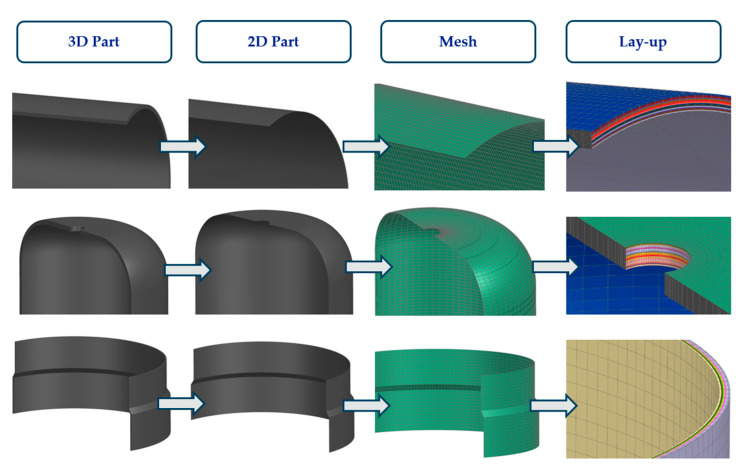
Visualization of dome, cylinder, and reinforcing band lay-up.

All components were fabricated using PHLP, with each unidirectional (UD) layer of CFRP placed in a metallic mold prior to assembly. Figure 7 highlights the development process from 3D geometry to 2D sections, mesh generation, and the application of composite layers, providing a detailed overview of how each composite layer is arranged in the metallic mold. The reference surface for this model is the bottom surface, corresponding to the first ply of the laminate. Different colors in the visualization represent varying ply angles, facilitating an understanding of the layer sequence and orientation during the PHLP.

#### 2.1.5. Loading Conditions

**Figure 8:**
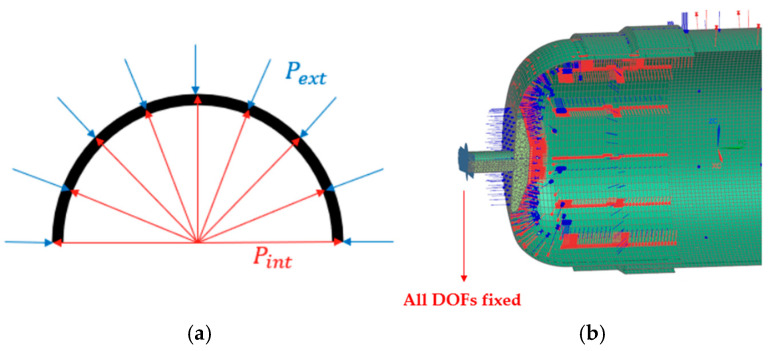
(**a**) Representation of internal and external pressure of CPV [27]; (**b**) Pressure loads and boundary conditions applied in the numerical model.

In both the numerical and physical tests of the CPV, pressure gradually increased from a low value until failure was observed. The load environment for the numerical model is carefully calibrated to replicate the conditions of the physical test, which is a hydraulic pressure test, ensuring that the model validation process is accurate. During hydraulic testing, the CPV is filled with water and placed inside a metallic testing chamber, also filled with water for safety reasons, which subjects the vessel to both internal and external hydrostatic pressures. In the virtual model, internal pressure is applied to the inner surfaces of each component (indicated in red), while external pressure, equal to atmospheric pressure, is applied to the outer surfaces (indicated in blue), as illustrated in Figure 8b [28].

#### 2.1.6. Boundary Conditions (BCs)

To ensure the accuracy and relevance of the virtual and physical model, it is essential to apply to virtual simulations similar BCs with physical tests. In this context, BCs refer to the constraints and contact type. In the physical test, the pressure vessel is secured at one polar opening, where it is fully supported in the valve of the test vessel’s cap. This setup constrains all degrees of freedom at this fixed polar opening. Conversely, the opposite polar opening of the vessel is allowed to deform and rotate freely in all directions, as illustrated in Figure 8b.

As for contact conditions, the interface between the cylinder and the domes involves an overlap that necessitates a robust bondline to withstand internal pressure. To reinforce this connection, adhesive is applied to the surfaces of both the cylinder and the domes that are connected. Since the vessel in this study is a prototype and will only be hydraulically tested, for cost and availability reasons, the performance of the adhesive in contact with hydrogen was not evaluated. In the virtual model, this bondline is simulated using the Surface-to-Surface Gluing feature available in the software. Surface-to-Surface Gluing is a commonly used feature of NX Siemens/Simcenter for simulating the bonding between two surfaces. This approach involves defining the contact surfaces and applying a glue connection to them. While this method does not allow for the specification of adhesive properties, it is suitable for this study’s purpose, which focuses on evaluating the overall structural integrity of the vessel rather than the detailed performance of the adhesive.

#### 2.1.7. Progressive Failure Analysis

Failure in composite materials can be categorized into several types, each arising from different mechanisms like matrix and fiber cracking, delamination, fiber bridging effect, fiber misalignment, and bundles thinning [6,29,30,31]. The failure of an individual lamina, referred to as the FPF, does not necessarily indicate structural failure, as the failure process in fiber-reinforced laminates is gradual and progressive [15]. The composite’s microstructure is complex and heterogeneous, which makes internal damage difficult to detect, as surface signs of failure may not be apparent [12]. As pressure increases, progressive failure modes such as interface debonding and matrix cracking become evident. According to the design requirements for composite pressure vessels, minimal matrix cracking is acceptable under operational pressures. In the progressive failure model described in this study, the failure of a single ply between the FPF and the ultimate burst pressure (UBP) may not lead to the failure of the entire structure or an accelerated failure rate, if the ply failure is local and not global. Obviously, if a ply fails in an extensive area of the structure, then delamination and global failure may occur due to the brittle nature of the failure. However, if the ply failure happens locally, then it is examined if the load may be safely redistributed to the sections right before and right after the critical section, which are intact and capable of carrying the load. This load transfer mechanism is examined by applying the load at the initial failed section after degrading the failed ply, making it inactive. If the load can be safely carried by the rest plies, then and only then, can it be safely transferred to the rest of the structure. So, when a lamina fails locally in a laminate, stress is redistributed among the remaining laminate. A composite vessel is considered to have failed when the maximum load level is reached, typically following multiple lamina failures, leading to UBP and a sudden appearance of extensive fiber breakage. This methodology provides a simplified approach where neither the specific failure mode nor the detailed progression of failure is analyzed. It serves as a foundational method for predicting ultimate UBP and can be a starting point for future research focused on predicting failure propagation in detail.

Numerical burst pressure results are heavily influenced by the failure criterion used. An extensive literature survey has identified several prominent failure criteria for composite laminates. The four widely used failure theories are as follows [6,11,12]:

- Maximum Stress Criterion
- Tsai-Hill Criterion
- Tsai-Wu Criterion
- Hashin Criterion

The Maximum Stress Criterion is often effective for isotropic materials and carbon fiber fractures. The Tsai-Hill Criterion considers strain energy and is suitable for orthotropic materials, though it assumes individual laminas to be isotropic. The Hashin Criterion is more appropriate for modeling matrix cracking, while the Tsai-Wu Criterion incorporates strength tensors and is applicable to a broader range of anisotropic materials [10,12].

Comparing these criteria shows that while the Tsai-Wu or Tsai-Hill criteria may exhibit discrepancies between experimental and numerical data, the Maximum Stress and Hashin criteria provide more accurate predictions of FPF. Among these, the Maximum Stress and Hashin criteria are preferred due to their ability to differentiate between failure modes such as fiber breakage, matrix cracking, and interfacial debonding, which is crucial for predicting laminate pressure failure [6,14].

Although the Tsai-Wu criterion is recognized for its accuracy in predicting failure pressure, it primarily identifies FPF and may not fully capture progressive failure. Given that this study aims to compare numerical and experimental results and considering that experimental tests will not provide data on burst failure modes, the Tsai-Wu criterion is selected for its accuracy in predicting FPF, despite its limitations in progressive failure analysis [10,11].

After selecting the Tsai-Wu failure criterion, the next step involves calculating the FPF. While FPF provides a conservative estimate, it is not sufficient for accurately predicting burst pressure, as the actual burst pressure typically exceeds the pressure at FPF. Although this conservative approach ensures safety during the design process, it can result in over-sizing of the vessel [10].

**Figure 9:**
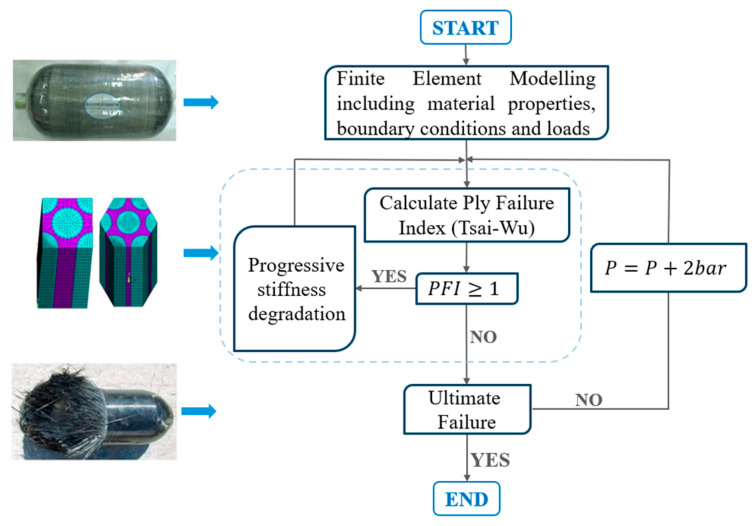
Development of progressive damage model [15,19,37].

To achieve a more precise prediction of the burst pressure, it is necessary to develop a progressive damage model that can accurately predict the last ply failure (LPF) pressure, which corresponds to the UBP of the composite vessel, so applied pressure is incrementally increased until LPF is reached. Predicting burst pressure is closely tied to numerical convergence issues in finite element analysis. Various studies have approached this challenge differently. Mao et al. [32] used statistical methods and experiments to estimate burst pressure, while Hwang et al. [33] employed probabilistic failure analysis to understand the effect of size on burst pressure. However, these studies did not address progressive failure properties. Hwang et al. [34] and Sun et al. [35] used nonlinear finite element analysis, incorporating the maximum stress failure criterion and a stiffness degradation model to predict burst pressure [15]. However, in this study, we will present a progressive damage model based on Rafiee and Torabi [14] and Roy and Tsai [36] research in the following steps and presented in Figure 9:

1. Analysis inputs
2. Stress analysis and Failure evaluation
3. Material degradation
4. Burst pressure detection

In Step 1, FEM incorporating the material properties, boundary conditions, and an initial internal pressure is established. Until the pressure reached a PFI close to 1, the pressure increase was rapid (ΔP = 10 bar). At 60 bar, the pressure increase was reduced to ΔP = 2 bar, and for a more precise calculation of critical pressures (PFI = 1), an increase of ΔP < 2 bar was applied [14]. In Step 2, the occurrence of failure is examined using the Tsai-Wu failure criterion, with PFI calculated for each ply. If no failure is detected (PFI < 1 for all plies), the pressure is increased, and the analysis continues. If a ply fails (PFI ≥ 1), we proceed to Step 3. Material degradation is the most critical step of this process. According to [14], various methods, such as the ply-discount method or continuum damage mechanics (CDM), can be employed in conjunction with commercial FE packages. In the ply-discount method, mechanical properties are suddenly degraded after failure, reducing properties to zero or a small fraction of the original value. However, this method cannot capture gradual degradation, which is more appropriate at high crack densities and relies on empirical values. Conversely, CDM accounts for the gradual degradation and correlates stiffness loss with internal damage state parameters [12]. In this study, however, the equipment used in the real test could not identify the failure type, limiting the application of a detailed model for comparison. Therefore, in Step 3, after Tsai-Wu was selected as the most accurate failure criterion for identifying FPF, a sudden degradation of mechanical properties after the occurrence of failure in each layer was implemented. More specifically, failed ply’s elastic properties are degraded, simulating an inactive ply by rendering it incapable of carrying any remarkable load. To ensure the failed ply is fully excluded from a possible unrealistic re-failure, as it is still able to carry minor loads, we artificially increase its tensile strength. The remaining load is then redistributed to the other intact plies and if no further failure occurs, pressure is increased until multiple plies fail simultaneously indicating the vessel’s burst, which is Step 4 [6,14,15].

### 2.2. Hydraulic Burst Pressure Test

According to [3,38], CPV certification testing comprises three phases: baseline verification, performance durability (hydraulic tests), and expected on-road performance (pneumatic tests). In this study, as the CPV is a prototype, a baseline verification test, specifically the Hydraulic Burst Pressure Test, will be conducted. Prior to testing, it is crucial to document the data presented in Table 5 to ensure an accurate evaluation of the results.

#### Hydraulic Burst Pressure Test Procedure

**Figure 10:**
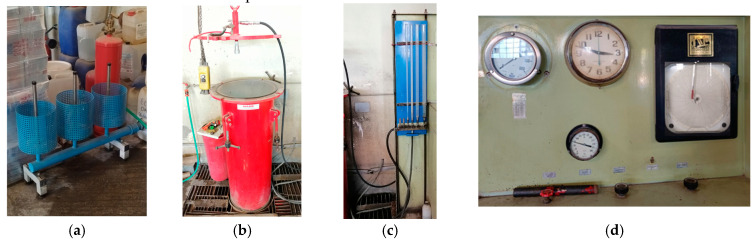
(**a**) Fluid filling station; (**b**) Metallic Testing vessel; (**c**) Water supply pipes and volumetric tubes; (**d**) Pressure control panel.

While some studies, such as [39], utilize liquid nitrogen (LN2) for burst testing, water at 15 °C is used in the baseline tests due to its superior safety profile. Water minimizes the energy released during a burst, making it a safer alternative compared to gases. Safety is the paramount consideration when conducting hydraulic pressure tests, as the potential for harm from cracked pieces during a vessel burst is significant. To mitigate these risks, the hydraulic test was performed within a metallic testing vessel filled with water, which served to contain any fragments from the burst explosion. Figure 10 outlines the experimental setup.

The experimental procedure follows the following steps:

- **Step 1: Preparation and Filling of the CPV**

The CPV is first placed inside one of the cylinders of Figure 10a. Water is then introduced into the CPV through a water supply pipe. This bottom-to-top filling process is crucial as it allows air to escape as the CPV fills with water. Once water overflows from the top valve, indicating that the vessel is fully filled, the CPV is sealed using a female metal block.

- **Step 2: Securing the CPV and Placement in the Metallic Testing Vessel**

After sealing, the CPV is secured by supporting it on the cap’s valve, preventing any movement in the one polar opening during loading. The CPV is then placed inside the metallic testing vessel of Figure 10b, which acts as the containment chamber for the test. The metallic testing vessel is a 3-m-long metallic container filled with water and equipped with an accessible opening cap, partially submerged in the ground for safety.

- **Step 3: Pressure Increase and Monitoring**

The testing team manually increases the pressure inside the CPV, while monitoring the pressure values using manometers. Water is supplied into the CPV via Water Supply Pipes (Figure 10c), with the first pipe directing water into the CPV during the pressure increase through the Pressure Control Panel (Figure 10d) and the second pipe connecting the testing vessel’s interior to the Volumetric Tubes (Figure 10c).

Following the completion of the preliminary steps, the hydraulic burst test started. Once the setup was prepared, the internal pressure gradually increased at a constant rate of 3.5 bar/s. Burst failure occurred at 91.7 bar, marked by a minor explosion that caused water to overflow from the slightly open cap of the testing vessel, along with a rapid drop of pressure. The burst pressure was recorded, and the vessel was carefully opened to safely transfer the burst tank for a detailed examination of the failure locations.

## 3. Results

### 3.1. Numerical Burst Pressure and Burst Locations

Following the progressive failure model developed in Section 2.1.7, the internal pressure of the tank was incrementally increased in steps of 2 bar. When a ply failed, identified by reaching PFI≥1, its material properties were degraded, according to the cross-section the failure was, to prevent it from carrying any load, and the simulation was rerun under the same loading conditions to observe load redistribution. The results of this iterative process are summarized in Table 6, Table 7 and Table 8, providing a detailed breakdown of the progressive failure analysis and including the pressures at which individual plies fail and their respective PFI values. To enhance clarity, the PFI values for plies are color-coded based on their failure status. Specifically, PFI values for plies currently failing are highlighted in red, while those for plies expected to fail are next in orange. For plies that have already failed, the keyword “FAILED” is appended to their PFI values. More specifically, Table 6 indicates FPF at 71 bars in ply 4, located at cross-section 1, specifically at the polar boss-dome interface. This region is particularly critical due to the material transition, resulting in a rapid change in stiffness and stress gathering. Further analysis of the PFI data reveals that degradation of ply 4 triggers failure in ply 3 at the same pressure and location. At 79 bar, as shown in Table 7, ply 12 fails at cross-section 2, where there is a change of thickness, another critical region due to material stiffness transitions. Ply 6 of cross-section 1 fails at 85 bar as the internal pressure increases further. At 89.5 bar, failure begins with ply 1 in cross-section 1. Degrading its properties and rerunning the simulation under the same pressure leads to additional failures: ply 2 and ply 8 in cross-section 1, with PFIs of 1.298 and 1.138, respectively, and ply 10 in cross-section 2, with a PFI of 1.005. The collective failure of multiple plies confirms that at 89.5 bar, the ultimate failure of the tank occurs establishing the NBP at 89.5 bar. Notably, failure occurs exclusively in cross-sections 1 and 2, with no failure observed in cross-section 3, as shown in Table 7.

Building on this data, Table 9 visually summarizes the vessel’s condition under critical pressure levels, highlighting instances of ply failure, the specific ply number, failure location, and associated PFI.

### 3.2. Experimental Burst Pressure and Burst Locations

**Figure 11:**
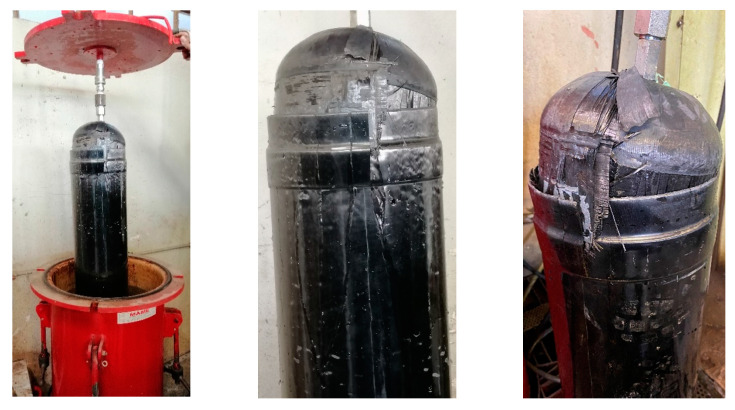
CPV after burst.

**Figure 12:**
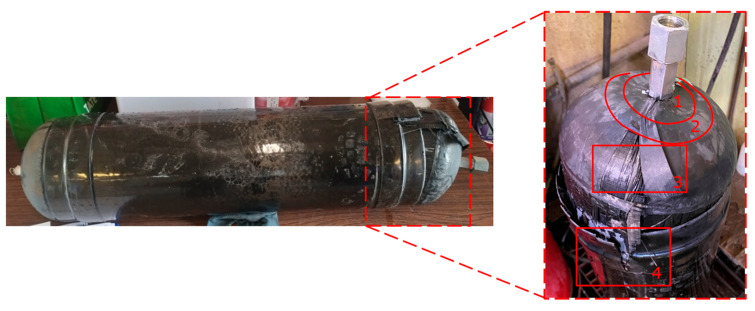
CPV visual inspection.

The hydraulic burst test produced significant results, demonstrating that the CPV designed in this study can withstand a pressure of 91.7 bar. However, the test did not provide detailed insights into the damage mechanisms involved, as nondestructive testing (NDT) was challenging to implement underwater. So, proceeding with visual inspection, it was revealed that there was damage at the polar boss-dome interface (Location 1) and in the change of thickness region (Location 2), leading to delamination in the dome (Location 3). Ultimately, this resulted in complete debonding and failure of the reinforcing band (Location 4) and the full detachment of one dome from the CPV, as shown in Figure 11 and Figure 12.

### 3.3. Comparison Between Virtual and Experimental Results

The experimentally determined burst pressure of this study’s CPV was 91.7 bar, while its predicted burst pressure was 89.5 bar. This yields a discrepancy of 2.3%, as calculated by the following formula:

$$\%error=\frac{\left|EBP-NBP\right|}{EBP}=\frac{\left|91.7\;\mathrm{bar}-89.5\;\mathrm{bar}\right|}{91.7\;\mathrm{bar}}=2.3\%\;\;\;\;$$

The observed 2.3% discrepancy between the NBP of 88.5 bar and experimental burst pressure (EBP) of 91.7 bar can be attributed to several factors related to the numerical model, the manufacturing process, and the stochastic nature of composite materials. Failure criterion and glue connection selected, non-conformances in manufacturing, and defects of materials can impact accuracy. In [12], the average difference between experimental and predicted results was reported to be 5.4%, validating the reliability of the method for calculating the elastic coefficients of stiffness degradation and the progressive damage model based on the Puck criterion. In our study, the average difference of 2.3% further confirms that the progressive failure method used to calculate the NBP of the CPV is reliable and closely aligns with the experimental results.

In the examination of numerical outcomes, we focus on critical pressures to compare the performance of the numerical and real models under specific pressure conditions. In Table 10, we conduct a comparative analysis of the numerical and real models to determine the extent to which the numerical model aligns with the actual behavior of the composite pressure vessel in critical pressures.

**Figure 13:**
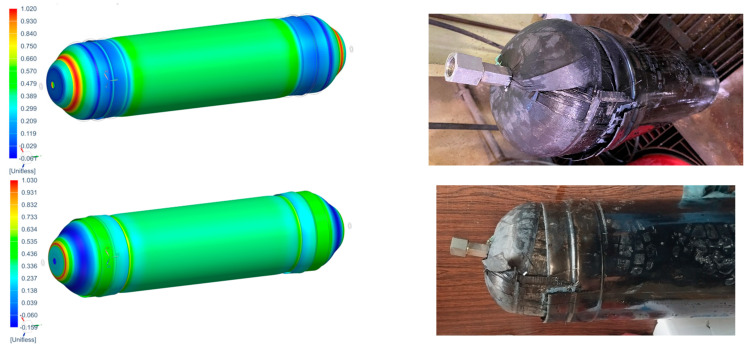
Comparison between numerical (PFI) and real model failure locations.

Regarding the failure locations of the CPV, Figure 13 demonstrates that the virtual model effectively predicts the general location of failure, which occurs in the fixed dome. However, while the model captures the primary failure location, it does not provide conclusive insights into the progression of damage or the hierarchy of failures. Moreover, Figure 13 highlights that the virtual model displays a uniform failure pattern, whereas the physical model reveals a more localized failure behavior. This discrepancy arises due to the idealized assumptions in the virtual model, which employs homogeneous material properties, resulting in a uniform stress distribution. In contrast, the physical model accounts for stochastic natural material variability, where localized inconsistencies in mechanical properties lead to stress concentrations and non-uniform failure patterns.

## 4. Conclusions

### 4.1. End-Product Quality Parameters

For this study, a CPV with a length of 795 mm (pole to pole), a diameter of 210 mm at the reinforcing band, and a total volume of 22 L was selected. From the numerical model, the FPF was calculated to occur at 71 bar, and the LPF was estimated at 89.5 bar. In contrast, the UBP obtained from the real model testing was 91.7 bar. Based on [26,39], a safety factor of 1.5 was selected, leading to the adoption of MEOP equal to 61.13 bar for the structural design which addresses the initial target to manufacture a tank that has MEOP equal to 60 bar. Table 11 presents the detailed geometry and performance outcomes of the CPV analyzed in this study.

In conclusion, this study successfully employed a cost and time-efficient analysis, along with a simplified progressive failure model, to predict the failure progression beyond the FPF of a Type V CPV. A robust numerical model was developed, capable of accurately predicting both the burst pressure and the burst location of Type V CPVs. Furthermore, this adaptable model can be applied to CPVs with varying pressure limits and hydrogen storage configurations, providing a valuable framework for advancing the design and testing of CPVs across a wide range of applications.

### 4.2. Future Development

Future work will focus on validating the proposed methodology by applying it to a wider range of CPV designs, as well as to simpler cylindrical structures, to comprehensively assess its robustness and reliability. Additionally, as this study primarily focused on the structural performance of a Type V CPV rather than its hydrogen storage capability and given that hydrogen permeability represents a critical challenge in hydrogen storage systems [40], future research will incorporate hydrogen testing and simulations to evaluate and predict permeability characteristics.

The successful validation of the design, numerical modeling, manufacturing, and testing processes in the prototype CPV establishes a solid foundation for scaling up hydrogen tank production to address diverse market demands. The developed methodologies can be adapted to produce Type V CPVs in various sizes, tailored for specific applications. Future developments will focus on industrializing the current tank design, integrating advanced manufacturing technologies such as AFP, and further refining the production process to enhance efficiency and scalability.

## Figures and Tables

**Table 1:** Different joint configurations.

Configuration		Joint Visualization
1	Simple	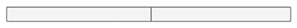
2	Single	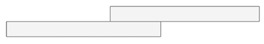
3	Double	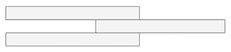
4	Scarf	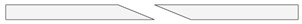
5	Stepped	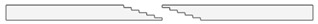

**Table 2:** Mesh independence study.

Mesh Size (mm)	Number of Elements	Ply Failure Index (PFI) for Ply 1	Time	Visualization
2	243,423	0.601	4 min 31 s	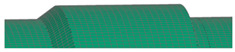
3	109,856	0.593	1 min 41 s	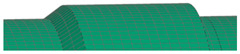
4	64,433	0.592	1 min 16 s	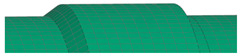
5	43,660	0.571	31 s	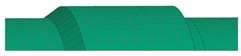
6	33,872	0.571	23 s	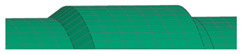
7	28,179	0.577	21 s	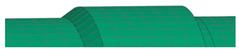

**Table 3:** State-of-the-art composite materials for CPVs.

Reference	Fiber	Manufacturer	Resin	Type of CPV
[6]	T300	Toray Industries, Tokyo, Japan	LY556+HT972	Type V
[21]	T800S 24 K	Toray Industries, Tokyo, Japan	UF3323	Type IV
[22]	T700-12K-50C	Toray Industries, Tokyo, Japan	UF3369	Type V
[23] *	T300	Toray Industries, Tokyo, Japan	934	Type V
[24] *	1200 Tex Fiber	Saint-Gobain Vetrotex, Courbevoie, France	A CY22	Type V
[25]	600 Tex Fiber	Saint-Gobain Vetrotex, Courbevoie, France	CY-225	Type V
[26]	T1000	Toray Industries, Tokyo, Japan	31-43B	Type III, Type IV
[27]	T300	Toray Industries, Tokyo, Japan	N5208	Type V
[20]	IM7	Hexcel, Stamford, Connecticut, USA	8552	Type V

* Cylindrical section.

**Table 4:** Mechanical properties of CF/E.

Material Property		Value	Unit
Tension	0° $\;\mathrm{Tensile}\;\mathrm{strength},\;\sigma_{t1}$	2354	MPa
	0° $\mathrm{Tensile}\;\mathrm{modulus},\;E_{1}$	116.6	GPa
	90° $\mathrm{Tensile}\;\mathrm{strength},\;\sigma_{t2}$	34.3	MPa
	90° $\;\mathrm{Tensile}\;\mathrm{modulus},\;E_{2}$	7.77	GPa
Compression	0° $\mathrm{Compressive}\;\mathrm{strength},\;\sigma_{c1}$	1102	MPa
	0° $\mathrm{Compressive}\;\mathrm{modulus},\;E_{1c}$	104.3	GPa
	90° $\;\mathrm{Compressive}\;\mathrm{strength},\;\sigma_{c2}$	184	MPa
	90° $\;\mathrm{Compressive}\;\mathrm{modulus},\;E_{2c}$	8.10	GPa
Shear	±45° $\;\mathrm{In}-\mathrm{Plane}\;\mathrm{shear}\;\mathrm{strength},\;\tau_{12}$	104.5	MPa
	±45° $\;\mathrm{In}-\mathrm{Plane}\;\mathrm{shear}\;\mathrm{modulus},\;G_{12}$	3.6	GPa
	0° $\;\mathrm{Interlaminar}\;\mathrm{shear}\;\mathrm{strength},\;\tau_{23}$	82.7	MPa
Poisson’s ratio	$\nu_{12}$	0.3	-

**Table 5:** Documentation of hydraulic burst pressure test inputs.

Hydraulic Test Inputs		
Structure	Weight	4.5 kg
	Size	795 mm × 210 mm
	Volume	22 L
Testing Environment	$T_{environment}$	17 °C
	Humidity	33%
	Location	Inside a metallic testing vessel
Fluid	Type of fluid	Water
	$T_{fluid}$	15 °C
Safety measures	Surrounding environment	All equipment is at a minimum distance of 3 m from the test location
	Surrounding test team	2 m distance from the test location, safety glasses
Loading conditions	Pressure ratio	3.5 bar/s

**Table 6:** Cross-section 1: PFI values from FPF-LPF.

		Cross-Section 1					
		71	71 (Stresses Redistribution)	85	85 (Stresses Redistribution)	89.5	89.5 (Stresses Redistribution)
		FPF	Failure	Failure	No additional failure	Failure	Ultimate Failure
		Ply 4— Failure	Ply 4—Failed Ply 3—Failure Ply 6—Expected Failure	Ply 4—Failed Ply 3—Failed Ply 6—Failure Ply 1—Expected Failure	Ply 4—Failed Ply 3—Failed Ply 6—Failed Ply 1—Expected Failure	Ply 4—Failed Ply 3—Failed Ply 6—Failed Ply 1—Failure Ply 2—Expected Failure	Ply 4—Failed Ply 3—Failed Ply 6—Failed Ply 1—Failed Ply 2—Failure Ply 8—Failure
**Ply ID**	**Ply deg**	**PFI**					
1	0	0.591	0.617	**0.890**	**0.923**	**1.002**	**FAILED**
2	0	0.523	0.546	0.783	0.813	**0.881**	**1.298**
3	45	**0.989**	**1.030**	**FAILED**	**FAILED**	**FAILED**	**FAILED**
4	90	**1.001**	**FAILED**	**FAILED**	**FAILED**	**FAILED**	**FAILED**
5	0	0.343	0.360	0.503	0.525	0.566	0.806
6	90	0.721	**0.746**	**1.021**	**FAILED**	**FAILED**	**FAILED**
7	0	0.248	0.261	0.356	0.373	0.400	0.546
8	90	0.466	0.486	0.663	0.687	0.730	**1.138**
9	0	0.197	0.213	0.262	0.283	0.299	0.355
10	90	0.341	0.347	0.446	0.453	0.480	0.631
11	0	0.168	0.183	0.225	0.244	0.258	0.244
12	90	0.329	0.333	0.418	0.422	0.447	0.524
13	0	0.141	0.154	0.190	0.207	0.219	0.209
14	90	0.320	0.324	0.401	0.406	0.430	0.495
15	−45	0.064	0.068	0.159	0.167	0.177	0.213
16	90	0.312	0.316	0.387	0.392	0.414	0.469
17	0	0.088	0.099	0.122	0.138	0.146	0.144

**Table 7:** Cross-section 2: PFI values from FPF-LPF.

Cross-Section 2				
		79	79 (Stresses Redistribution)	89.5 (Stresses Redistribution)
		Failure	No additional failure	Ultimate Failure
		Ply 12—Failure Ply 10—Expected Failure	Ply 12—Failed Ply 10—Expected Failure	Ply 12—Failed Ply 10—Failure
**Ply ID**	**Ply deg**	**PFI**		
1	0	0.681	0.803	0.950
2	90	0.720	0.788	0.931
3	0	0.644	0.759	0.892
4	90	0.546	0.591	0.774
5	0	0.613	0.722	0.844
6	90	0.405	0.407	0.799
7	0	0.595	0.701	0.815
8	90	0.536	0.542	0.669
9	0	0.588	0.692	0.804
10	90	**0.764**	**0.779**	**1.005**
11	0	0.587	0.691	0.803
12	90	**1.020**	**FAILED**	**FAILED**
13	0	0.587	0.691	0.801

**Table 8:** Cross-section 3: PFI values from FPF-LPF.

Cross-Section 3		
		89.5 (Stresses Redistribution)
		No Failure
		13 plies
**Ply ID**	**Ply deg**	**PFI**
1	0	0.717
2	60	0.771
3	90	0.825
4	−60	0.64
5	90	0.662
6	90	0.583
7	0	0.576
8	90	0.505
9	90	0.524
10	−60	0.538
11	90	0.562
12	60	0.568
13	0	0.585

**Table 9:** Numerical results of CPV.

**P = 71 bar** Ply 4 PFI = 1.001 Cross-section 1	**P = 71 bar** Ply 3 PFI = 1.03 Cross-section 1		**P = 79 bar** Ply 12 PFI = 1.02 Cross-section 2
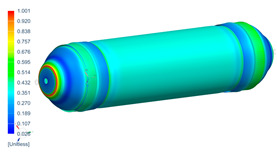	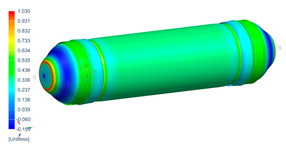		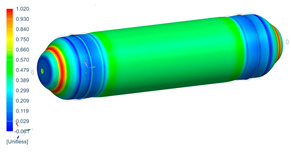
**P = 85 bar** Ply 6 PFI = 1.021 Cross-section 1	**P = 89.5 bar** Ply 1 PFI:1.002 Cross-section 1		**P = 89.5 bar** Ply 2 PFI:1.298 Cross-section 1
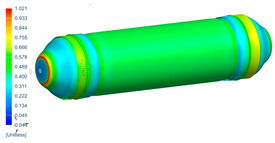	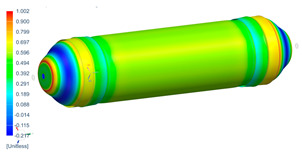		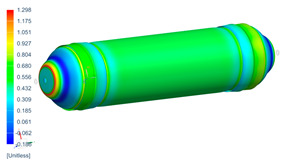
**P = 89.5 bar** Ply 8 PFI = 1.138 Cross-section 1		**P = 89.5 bar** Ply 10 PFI = 1.005 Cross-section 2	
** 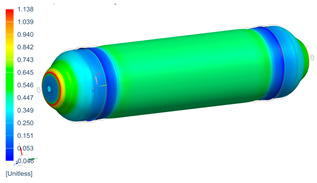 **		** 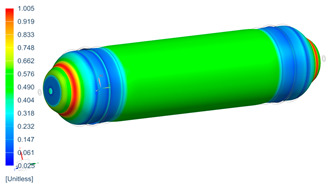 **	

**Table 10:** Comparative analysis of numerical and real model performance under critical pressures.

Critical Pressures (bar)	Numerical Results	Experimental Results
61	Initial Critical Pressure	
	PFI < 1 → No failure Max PFI = 0.741 → Ply 4	No visual failure up to 61 bar.
71	FPF of virtual model	
	PFI > 1 → Failure Max PFI = 1.001 → Ply 4 → FPF	No visual failure occurred up to 71 bar.
89.5	NBP	
	PFI > 1 → Failure 4 plies fail together (ply 1, 2, 8, 10) → Ultimate Failure	No visual failure occurred up to 89.5 bar
91.7	Experimental burst pressure (EBP)	
	Burst has already occurred	Burst explosion

**Table 11:** End-product quality parameters.

Parameters		Value
Geometry	Size (Length × Diameter)	795 mm × 210 mm
	Tank weight	4.5 kg
	Tank capacity	22 L
Performance	Safety factor	1.5
	Numerical burst pressure (NBP)	89.5 bar
	Experimental burst pressure (EBP)	91.7 bar
	%error	2.3%
	Maximum expected operating pressure (MEOP)	$\frac{91.7}{1.5\;}=61.13\;\mathrm{bar}$

## Data Availability

The original contributions presented in this study are included in the article. Further inquiries can be directed to the corresponding author.
